# Repetitive non-typhoidal *Salmonella* exposure is an environmental risk factor for colon cancer and tumor growth

**DOI:** 10.1016/j.xcrm.2022.100852

**Published:** 2022-12-20

**Authors:** Daphne M. van Elsland, Janneke W. Duijster, Jilei Zhang, Virginie Stévenin, Yongguo Zhang, Lang Zha, Yinglin Xia, Eelco Franz, Jun Sun, Lapo Mughini-Gras, Jacques Neefjes

**Affiliations:** 1 Department of Cell and Chemical Biology, Oncode Institute, Leiden University Medical Center (LUMC), 2333 Leiden, the Netherlands; 2 Center for Infectious Disease Control, National Institute for Public Health and the Environment (RIVM), 3721 Bilthoven, the Netherlands; 3 Division of Gastroenterology and Hepatology, Department of Medicine, Department of Microbiology/Immunology, University of Illinois Chicago, Chicago, IL 60612, USA; 4 UIC Cancer Center, University of Illinois Chicago, Chicago, IL 60612, USA; 5 Institute for Risk Assessment Sciences, Utrecht University, 3584 Utrecht, the Netherlands

**Keywords:** colon cancer, *Salmonella*, seroincidence, environmental risk factor

## Abstract

During infection, *Salmonella* hijacks essential host signaling pathways. These molecular manipulations disrupt cellular integrity and may induce oncogenic transformation. Systemic *S.* Typhi infections are linked to gallbladder cancer, whereas severe non-typhoidal *Salmonella* (NTS) infections are associated with colon cancer (CC). These diagnosed infections, however, represent only a small fraction of all NTS infections as many infections are mild and go unnoticed. To assess the overall impact of NTS infections, we performed a retrospective serological study on NTS exposure in patients with CC. The magnitude of exposure to NTS, as measured by serum antibody titer, is significantly positively associated with CC. Repetitively infecting mice with low NTS exposure showed similar accelerated tumor growth to that observed after high NTS exposure. At the cellular level, NTS preferably infects (pre-)transformed cells, and each infection round exponentially increases the rate of transformed cells. Thus, repetitive exposure to NTS associates with CC risk and accelerates tumor growth.

## Highlights

• Prospective colon cancer patients have a higher non-typhoidal *Salmonella* seroincidence
• Repetitive low exposure to non-typhoidal *Salmonella* is associated with colon cancer risk
• Repetitive infection with non-typhoidal *Salmonella* increases cancer growth

## Graphical abstract

**Figure:**
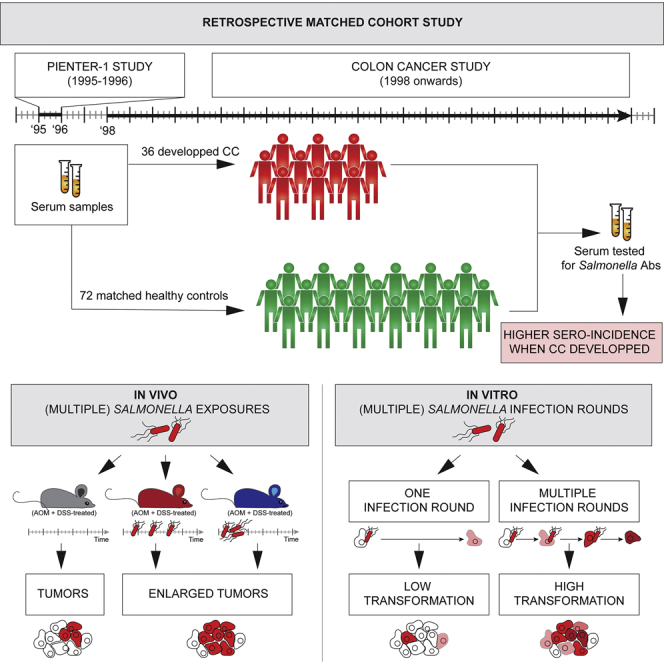

## Teaser

Van Elsland et al. observe a higher non-typhoidal *Salmonella* seroincidence among prospective colon cancer patients and an oncogenic role of repetitive non-typhoidal *Salmonella* infections in tissue culture and mouse models. These findings raise the importance of considering non-typhoidal *Salmonella* an environmental risk factor for colon cancer.

## Introduction

*Salmonella enterica* subspecies *enterica* is a Gram-negative bacterium including more than 2,500 different serovars that can cause gastrointestinal disease and occasionally invasive infection of variable severity. These serovars are commonly divided into two groups. The typhoidal serovars (i.e., Typhi and Paratyphi) are human-restricted pathogens that can cause the severe systemic illnesses typhoid or paratyphoid fever. The non-typhoidal *Salmonella* (NTS) serovars, of which Enteritidis and Typhimurium are among the most common ones in clinical patients, can colonize asymptomatically a broad range of animals and usually cause gastroenteritis in humans. As *S*. Typhi and Paratyphi are mainly transmitted between humans via the fecal-oral route, the vast majority of (para)typhoid fever cases occur in densely populated areas lacking access to improved sanitation.^1^ Conversely, NTS infections occur worldwide, are common in developed countries, and are transmitted mostly from animals to humans via food, as well as directly via animal contact or indirectly via the environment.^2^^,^^3^

Both typhoidal and non-typhoidal serovars have been linked to human cancer. Globally, the incidence of typhoid fever and gallbladder cancer (GBC) shows substantial geographical overlap.^4^^,^^5^ This link is further supported by histological findings of *S*. Typhi in tumors of patients with GBC from geographic areas with high GBC prevalence.^6^ Similar to *S*. Typhi, severe NTS infection is epidemiologically associated with increased colon cancer (CC) risk.^7^ Indeed, in a large registry-based nationwide cohort study in the Netherlands, the risk of proximal CC was twice as high among people with a laboratory-diagnosed NTS infection when compared with the general population.^7^

During host cell invasion, *Salmonella* injects over 30 different effector proteins into its host to increase its uptake, intracellular survival, and egress.^8^ Among these effectors, AvrA, SopE, SopE2, SopB, and SptP are known to mediate activation of the host’s β-catenin, MAPK, and AKT signaling pathways, respectively.^6^^,^^9^^,^^10^^,^^11^ The activation of these pathways by *Salmonella* results in transformation of both *in vitro* and *in vivo* models harboring pre-transformed genotypes, such as partial (heterozygote) or total (homozygote) deficiency of the tumor-suppressor genes Apc or Arf, respectively, and constitutive expression of the protooncogene c-MYC.^6^ Inhibition of *Salmonella*-activated pathways upon inhibitor treatment abrogated *Salmonella*-mediated transformation.^6^ Moreover, *Salmonella* mutants that lack T3SS (ΔprgH) failed to induce colon carcinomas in Apc heterozygous mice and showed a strong and significant reduction in their capacity to induce transformation in pre-transformed (*Arf*^−/−^, c-MYC) mouse embryonic fibroblasts.^6^ The activation of oncogenic pathways by *Salmonella* thus seems to primarily occur in a *Salmonella*-virulence-factor-dependent manner and, as such, contributes to one or more steps in the multi-step oncogenic transformation of cells.^6^^,^^12^^,^^13^

The severity of a *Salmonella* infection is determined by (1) host factors, (2) the *Salmonella* virulence profile, and (3) the number of Salmonellae ingested.^14^ While about 90,000 human salmonellosis cases are reported to public health authorities in Europe each year,^15^ this number is based on only those cases needing medical attention, laboratory diagnosis, and reporting to public health authorities. It has been estimated that, on average, for every reported salmonellosis case in Europe, approximately 57 *Salmonella* infections go unreported.^15^ The reported cases therefore represent mostly severe infections, i.e., a small fraction of all *Salmonella* infections occurring in the population. This has been further supported by serological studies where the rate of the immune-response-eliciting exposures to NTS was measured, showing that such exposure vastly exceeds the incidence of clinically overt salmonellosis, with people acquiring numerous mild NTS infections throughout their life.^16^^,^^17^^,^^18^

As severe or long-lasting *Salmonella* infections may promote colon carcinogenesis by virtue of their higher chance of affecting pre-transformed cells,^19^ we also have to consider repetitive exposure to NTS as contributing to the multi-step CC formation process. To test this, we integrated a serological and epidemiological approach with both *in vivo* and *in vitro* analyses, showed that the magnitude of exposure to NTS is associated with CC formation, and also showed that *in vivo* exposure to repetitive low doses of NTS contributes to CC in a similar manner as a single high NTS dose. We furthermore report that repetitive NTS infections significantly increase the proliferation of transformed cells in tissue culture experiments. As exposure to NTS is difficult to avoid, these results suggest that *Salmonella* should be considered an environmental risk factor for CC development.

## Results

### Increased *Salmonella* seroincidence is associated with increased CC risk

Previously we showed that reported *Salmonella* infections are epidemiologically associated with increased CC risk.^7^ However, this study included only reported *Salmonella* infections, which represent a small fraction of all NTS infections that people can acquire throughout life.^16^^,^^18^^,^^20^ To assess the risk of CC development as a function of the magnitude of NTS exposure, regardless of disease severity, we performed a retrospective matched cohort study on two linked datasets. The first dataset was derived from a nationwide cross-sectional serological survey conducted in the Netherlands between October 1995 and December 1996, the so-called “PIENTER-1” study.^18^ This study established a large serum bank with accompanying epidemiological data representative of the Dutch general population primarily aimed at immunosurveillance to evaluate the national immunization program. This source of information allowed us to establish, retrospectively, a cohort of people representative of a national population (and not limited to, for example, a hospital or GP cohort) with determined exposure levels to *Salmonella* based on serological testing performed at the beginning of a long follow-up period of up to 19 years to observe any CC diagnosis. The second dataset, obtained from the Netherlands Cancer Registry (NCR), covers all Dutch residents and includes data on patients diagnosed with CC in the proximal part of the colon since 1998 (ICD-10 codes: C180–C185). We focused our analyses on these colon subsites as our previous study highlighted a significantly increased risk of cancer after NTS infection only in the proximal, not the distal, part of the colon.^7^ The national population registry-based nature of both PIENTER-1 and NCR datasets coupled with the retrospective study design rendered the cohort virtually free from subject selection and loss to follow-up biases.

**Figure 1:**
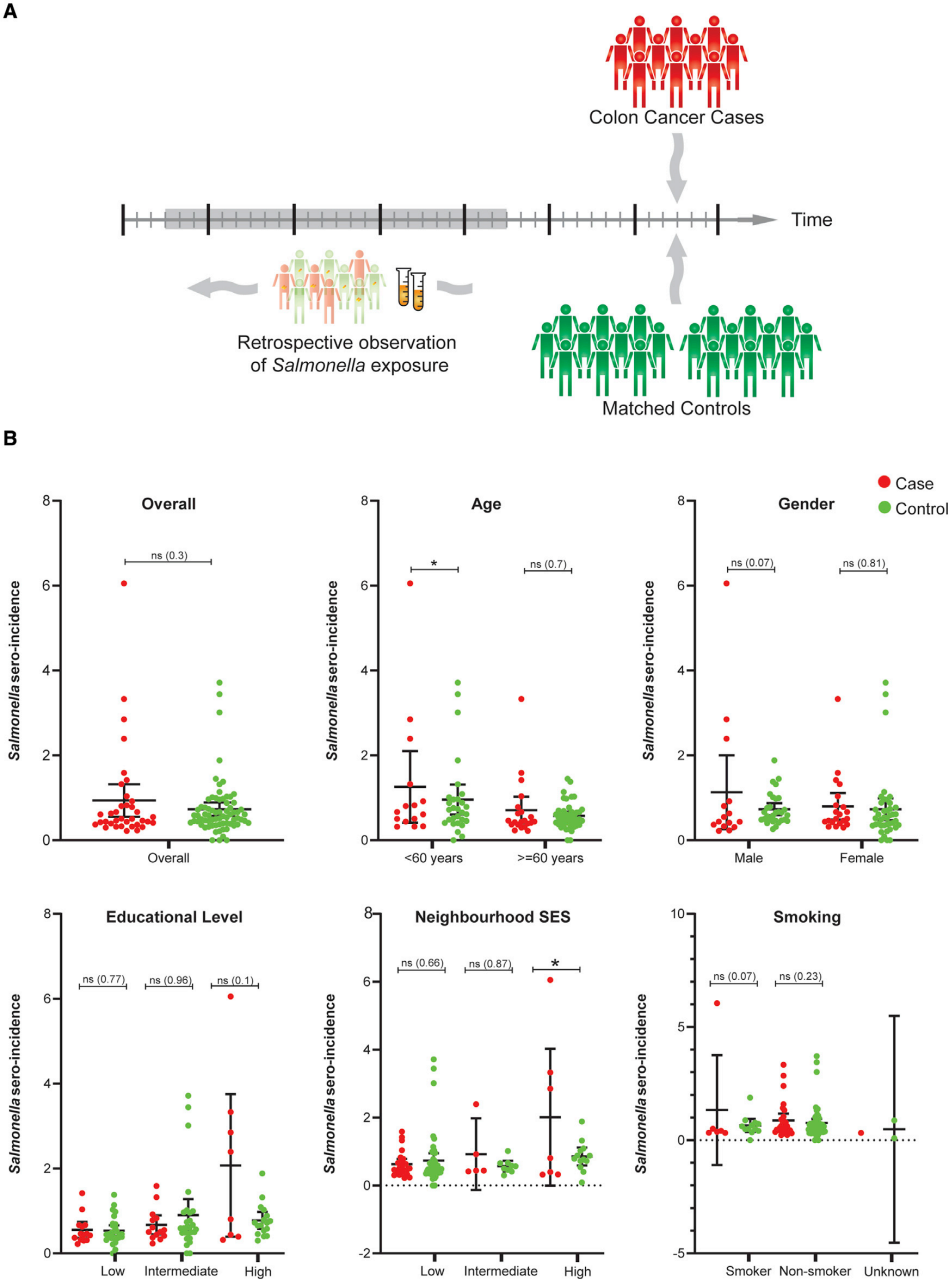
Increased seroincidence rates among individuals <60 years of age at serum sampling are significantly associated with increased CC risk (A) Schematic overview of study design: 36 participants in the PIENTER-1 study with a diagnosed cancer in the proximal colon were demographically matched at a 1:2 ratio with other PIENTER-1 study participants who were not diagnosed with CC (i.e., “controls”). The serum samples were tested for anti-*Salmonella* IgA, IgM, and IgG concentrations. (B) Concentrations of IgA, IgG, and IgM anti-*Salmonella* antibodies in cases and controls expressed in optical density (OD) values. (C) *Salmonella* seroincidence rates and colon cancer risk stratified by gender, age, ethnicity, educational level, socioeconomic status, andsmoking. ns, not significant; the value between brackets shows the p value of the corresponding hazard ratio. ∗p < 0.05.

By linking the PIENTER-1 study data to CC diagnoses in the NCR data, 36 participants in the PIENTER-1 study were found to have a diagnosed cancer in the proximal colon in the period between January 1^st^, 1998 and December 31^st^, 2017 (end of the present study period). Each of the 36 patients with CC was then demographically matched with two other PIENTER-1 study participants who were not diagnosed with CC (and were still alive) during the study period (Figure 1A). The characteristics of the total cohort are shown in Table S1. The cohort comprised 108 participants (36 CC cases and 72 CC-free individuals), consisting of 42% men and 58% women with a median age of 63 years (mean 60 years, interquartile range [IQR] 52–68 years) at serum sampling within the PIENTER-1 study.

The serum samples of the 36 patients with CC (i.e., “case”) and the 72 persons without CC (i.e., “controls”) were retrieved from the PIENTER-1 serum bank and tested for anti-*Salmonella* immunoglobulin A (IgA), IgM, and IgG concentrations using a validated mixed ELISA based on commercially available lipopolysaccharides of the two most common serovars, *S*. Enteritidis (O-antigens 1,9,12) and *S*. Typhi (O-antigens 4,5,12). These were used as capture antigens in solid phase and have been extensively validated as a means to determine the rate of a *Salmonella* infection.^21^ For each sample, the concentrations of each Ig isotype were measured and expressed as optical density (OD) units (Figure 1B). These OD values were then used to estimate the seroincidence of NTS infection, i.e., the average number of NTS infections per person per year, as a measure of NTS infection pressure or force of infection in the person in question. This was done considering the established kinetics of anti-*Salmonella* IgG, IgM, and IgA serum antibody levels following NTS infection^16^^,^^17^^,^^18^^,^^20^ using an established Bayesian back-calculation model available as an R package called “seroincidence.”^16^^,^^17^^,^^20^ The overall seroincidence was found to be 0.80 (95% confidence interval [CI] 0.62–0.98) NTS infections per person year. When stratified by CC status, the mean seroincidence was 0.94 (95% CI 0.55–1.32) NTS infections per person year among those who later developed CC, which is higher than the seroincidence of 0.73 (95% CI 0.57–0.89) NTS infections per person year in the control group. This difference was, however, not statistically significant (hazard ratio [HR] 1.24, 95% CI 0.82–1.88, p = 0.302) (Figure 1C, overall; Table S2).

In our previous study, the increased CC risk concerned specifically individuals age <60 years at reported NTS infection as CC risk increases significantly with age because of a multitude of other factors that may dilute the relatively smaller contribution of NTS infection.^7^ We therefore stratified the present analysis by age at serum sampling and found that increased seroincidence among individuals <60 years of age at serum sampling was significantly associated with increased CC risk (HR 1.41, 95% CI 1.03–1.94, p = 0.033) (Figure 1C, age; Table S2). Other factors like gender, educational level, or smoking were not significantly associated with increased seroincidence and CC risk (Figure 1C, gender, educational level, smoking; Table S2). The only other factor modifying significantly the effect of increased seroincidence on CC risk was living in neighborhoods of high socioeconomic status (SES) (HR 1.32, 95% CI 1.03–1.69, p = 0.027), suggesting that the link between NTS seroincidence and CC risk can be enhanced by additional environmental settings (Figure 1C, neighborhood SES; Table S2). In conclusion, the serological analyses indicated that a high NTS infection pressure (as defined by seroincidence) in age groups in which age itself can be expected to exert lower oncogenic effects than later in life may act as a risk factor for proximal CC.

### Impact of repetitive low dose NTS infections on CC formation in mice

To evaluate whether repetitive NTS infections are capable of triggering cell transformation *in vivo*, a mouse study was designed to compare their role in CC formation of repetitive mild infections versus a single severe infection. Since the severity of a NTS infection is determined by the NTS genotype, host factors, and ingested dose,^14^ we selected the optimal NTS strain for this long-term CC mouse study through an *in vivo* mortality and morbidity screen of several human clinical NTS isolates (Figure S1). Higher NTS doses are reported to give higher attack rates and more severe disease.^14^ Mild infections were thus mimicked by infecting mice with a low NTS inoculum of 10 bacteria, whereas a severe infection was mimicked by infecting mice with a high inoculum of 10,000 bacteria, a known and well-established dose of Salmonellae for mouse studies.^22^^,^^23^

**Figure 2:**
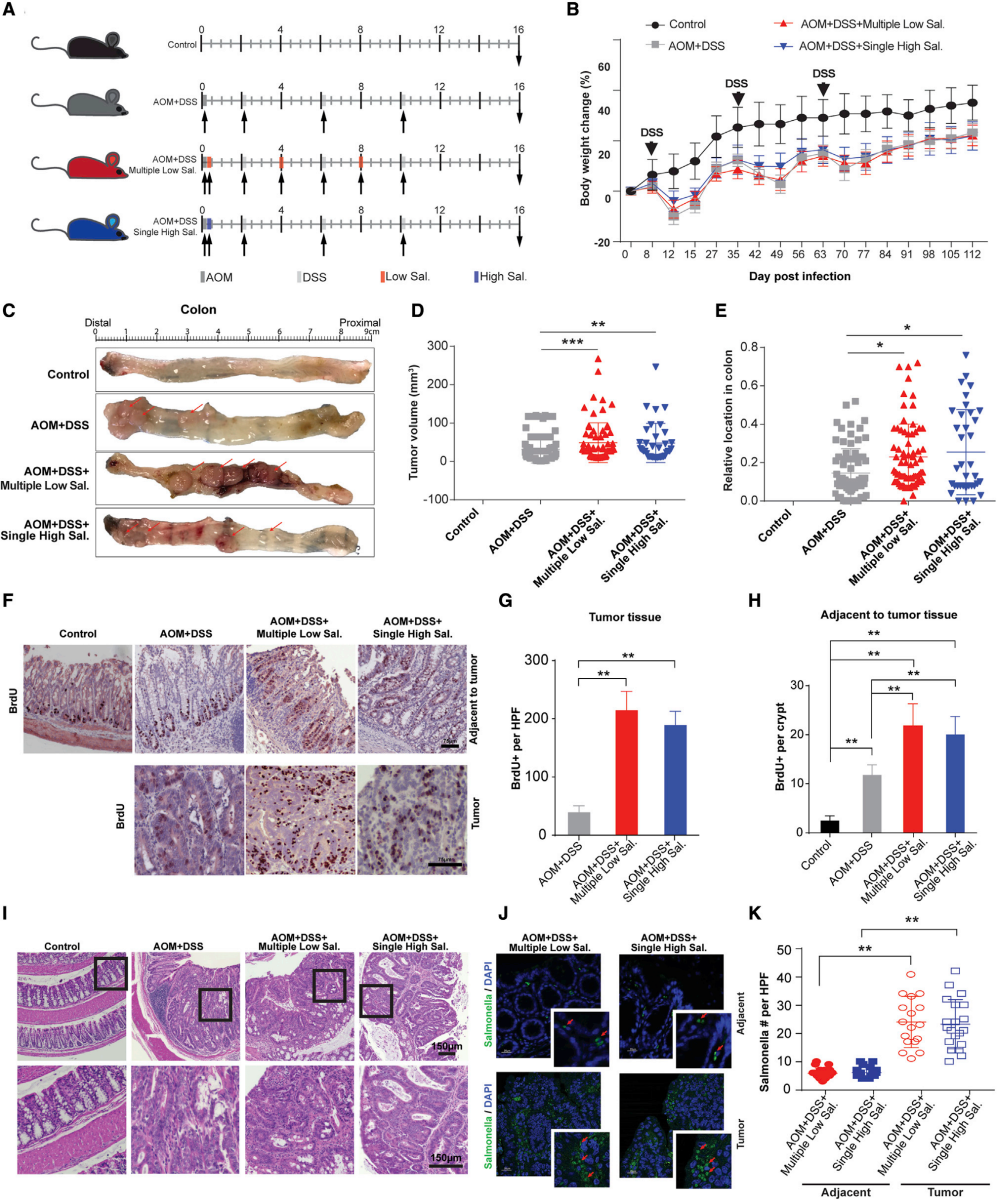
Repetitive low-dose NTS exposures have tumorigenic impact on colon cancer formation *in vivo* (A) Treatment timeline of mouse cohort. Mice were infected with either 10 CFUs (100-μL suspension in HBSS) for the repetitive low dose of *S*. typhimurium, with 10,000 CFUs (100-μL suspension in HBSS) for one single high dose or treated with sterile HBSS (control and AOM + DSS groups) by oral gavage at day 1. The carcinogen AOM was administrated through intraperitoneal injection at day 2 for all groups except for the control group. After a 7-day recovery period, the inflammatory agent dextran sodium sulphate (DSS) was administrated at 2% in drinking water for 7 days for all groups except for the control. This DSS treatment was repeated at 5 and 9 weeks. In case of repetitive low-dose infections, oral gavage with 10 CFUs (100-μL suspension in HBSS) of *S*. typhimurium was repeated at 4 and 8 weeks (Figure 2A). The experiment was evaluated at 16 weeks post infection. n = 10, 30, 31, and 29 for control, AOM + DSS, AOM + DSS + multiple low Sal., and AOM + DSS + single high Sal. groups, respectively. (B) Percentage of body weight change throughout the experiment for indicated groups of the mouse cohort. Data are expressed as mean ± SD. (C) Colonic tumors *in situ*. Representative colons of indicated groups of the mouse cohort at 16 weeks post infection. Tumors are indicated by red arrows. (D) The tumor volume of indicated groups within the mouse cohort. The data are expressed as mean ±SD; Kruskal-Wallis test, one-way ANOVA, ∗∗p < 0.01, ∗∗∗p < 0.001. (E) The tumor distribution of indicated groups within the mouse cohort. The relative location in the colon was defined as the distance from tumor to anus/colon length. The data are expressed as mean ± SD; Kruskal-Wallis test, one-way ANOVA, ∗p < 0.05. (F) Immunohistochemistry staining of BrdU in normal colon and colonic tumors of the mouse cohort. Scale bars, 75 μm. (G) Quantification of BrdU staining in tumors from the indicated groups in the mouse cohort. The data are expressed as mean ± SD; one-way ANOVA, ∗∗p < 0.01, n = 6 per group. (H) Quantification of BrdU staining in normal tissues from the indicated groups in the mouse cohort. The data are expressed mean ± SD; one-way ANOVA, ∗∗p < 0.01, n = 6 per group. (I) Representative images of normal control colon tissue and colon tumor tissues of indicated groups of the mouse cohort. The control tissue section is from different parts of the colon of control mice. The box shows the area of the zoom in in the bottom panel. Scale bars, 150 μm. (J) NTS invasion in the colon tissue. Localization of NTS (red arrow) in adjacent normal tissue and colonic tumor tissue was assessed by immunofluorescence staining with an NTS-specific antibody. (K) Quantification of NTS invasion in the colon tissue. The number of NTS was counted per high pure field (HPF). Data are expressed as mean ± SD; one-way ANOVA, ∗∗∗p < 0.001, n = 5–6 per group.

Mouse experiments were performed using specific-pathogen-free female C57BL/6 mice in a carcinogen azoxymethane (AOM)+ inflammatory agent dextran sodium sulphate (DSS) CC model, which has been extensively used as a model system to investigate the accelerating effect of NTS infections on the multi-step CC formation process.^24^ Single high-dose exposures (“single high Sal.”) were performed with single subjection to 10,000 bacteria (equivalent colony-forming unit [CFU]) in a 100-μL HBSS suspension. Repetitive low-dose exposures (“multiple low Sal.”) were performed with 3 subjections to 10 bacteria (equivalent CFU) in a 100-μL HBSS suspension. In case of repetitive infections, there were two 4-week intervals between exposures (weeks 1, 4, and 8). As control, non-infected untreated mice were used, as well as non-infected AOM + DSS-treated mice (Figure 2A).

Throughout the experiment, changes in body weight were monitored for all groups (Figure 2B). From week 0 to 16, overall weight increased for all four groups. The increased rates were, however, markedly different between the untreated control group and the AOM + DSS- and AOM + DSS-NTS-infected treatment groups. However, among the three treatment groups, no significant differences were observed. In the first 2 weeks after treatment initiation, the increasing rates of mouse body weight from the three treatment groups all slowed down. Until week 2 or 3, the average weight of all three treatment groups was significantly lower than the control group (Figure 2B). As no additional effects on body weight could be observed for any of the NTS exposures, we concluded that the observed effects on body weight changes were the result of AOM + DSS treatment only.

As anticipated from previous studies, AOM + DSS treatment was dominant for tumor formation.^24^ Colonic tumors were found to be formed at a similar incidence throughout all treatment groups with no significant differences in incidence in the case of AOM + DSS (76.67%), AOM + DSS + multiple low Sal. (70.97%) and AOM + DSS + single high Sal. (62.07%) (Figures 2C and S2). No tumors were formed in the control group (0/10) (Figures 2C and S2). Yet, tumor sizes of the mice in the groups exposed to multiple low (p < 0.001) or single high (p < 0.01) doses of NTS were significantly increased compared with the AOM + DSS-treated group (Figures 2C and 2D). NTS infection thus appears to accelerate tumor growth in this model, and multiple low doses of NTS trigger a similar tumorigenic impact on CC formation as a singular high dose of NTS. Moreover, the location of the colonic tumors was distributed from distal to proximal colon in both multiple low (p < 0.05) and single high (p < 0.05) NTS-exposed groups compared with the AOM + DSS-treated group, where the tumors were restricted to the distal colon (Figure 2E).

To further assess this attributing effect, colon tissues were subjected to hematoxylin and eosin (H&E) staining and pathology (Figure 2I). Lesions of colon tissues of AOM + DSS-treated mice revealed low-grade dysplasia in the formed tumors. In comparison, both the multiple-low-dose and single-high-dose NTS-exposed mouse tissues showed high-grade dysplasia. More specifically, a representative section of a tumor in the colon of AOM + DSS mice shows a mucosal lesion with low-grade dysplasia showing minor gland distortion and nuclear pseudostratification without significant atypia. Some normal colon tissue is still visible at the edge of the lesion. Representative sections through tumors of both the single-high and multiple-low NTS-exposed mice show high-grade dysplasia with mayor gland distortion, cribriform growth, and intraluminal cell debris. There is more obvious nuclear atypia with pseudostratification and hyperchromasia (Figure 2I). Control mice did not show any abnormalities (Figure 2I). We then evaluated tumor cells and their adjacent tissue growth by BrdU labeling. BrdU labeling was significantly higher in the tumors from both NTS-infected groups compared with the AOM + DSS control group (p < 0.01), with no significant difference between the single-high-dose and multiple-low-dose NTS-exposed groups (Figures 2F and 2G). In the tissue adjacent to the tumor, BrdU-positive cells in the colon crypts were significantly higher for all treatment groups compared with the non-treated control group (p < 0.01). Furthermore, both the low- and high-dose NTS-exposed mice displayed significantly higher BrdU signals in colon crypts than the AOM + DSS control group (p < 0.01) (Figures 2F and 2H). Similar to the tumor tissue, no significant difference in BrdU intensity between the singular and repetitive NTS-exposed mice were observed in the colon crypt tissue (Figures 2F and 2H). These data suggest that both repeated low-dose NTS infections and a single high-dose NTS infection accelerate proliferation of tumor and tumor-adjacent tissue. Colon tissues of mice exposed to both low-dose NTS infections and a single high-dose NTS infection were found to be colonized by NTS (Figure 2J). Tumor tissues were, however, colonized with significantly more bacteria compared with adjacent, non-tumor tissues (Figure 2K), indicating that in the case of both low and high inoculates, NTS preferentially accumulated in tumor tissues.

Since the AOM/DSS mouse model does not model sporadic CC development, we also tested the impact of NTS infection on CC formation in an APC conditional deficiency sporadic cancer mouse model. Due to the nature of this model, which does not allow for a longitudinal experiment due to the swift spontaneous tumor formation, a condition with (longitudinal) repetitive NTS infection could unfortunately not be tested. To this end, APC^ΔCEC^ mice and APC^15lox^ mice^25^^,^^26^ were subjected to only a single high-dose NTS exposure (“single high Sal.”) or were left untreated (“control”). Throughout the experiment, body weight changes were monitored for all groups. Although the body weight in the NTS-infected groups slowed down in the first 2 weeks after infection, no significant differences could be observed between the overall body weight of the control and single high Sal. groups (Figure S3A). Colonic tumors were formed in both APC^ΔCEC^ control and APC^ΔCEC^ single high Sal. groups (Figure S3B). Tumors in the APC^ΔCEC^ NTS-exposed group (APC^ΔCEC^ single high Sal.) were, however, significantly enlarged compared with the APC^ΔCEC^ control group (Figures S3B and S3C). NTS infection thus appears to also accelerate tumor growth in this model. Moreover, similar to the NTS-exposed groups in the AOM/DSS model, the location of the colonic tumors in the NTS-exposed APC^ΔCEC^ group was more distal compared with the APC^ΔCEC^ control group (Figures S3B and S3D). Spleen weights were, furthermore, found to be significantly increased after NTS infection. However, since no difference could be observed between APC^15lox^ control and APC^ΔCEC^ groups, inflammatory modulators are unlikely to play an important role in the NTS-accelerated tumor formation in the APC^ΔCEC^ mouse model (Figure S3E). Overall, these results indicate that NTS infection also appears to accelerate CC growth in a sporadic cancer mouse model.

### Repetitive NTS infection accelerates growth of pre-transformed cells

**Figure 3:**
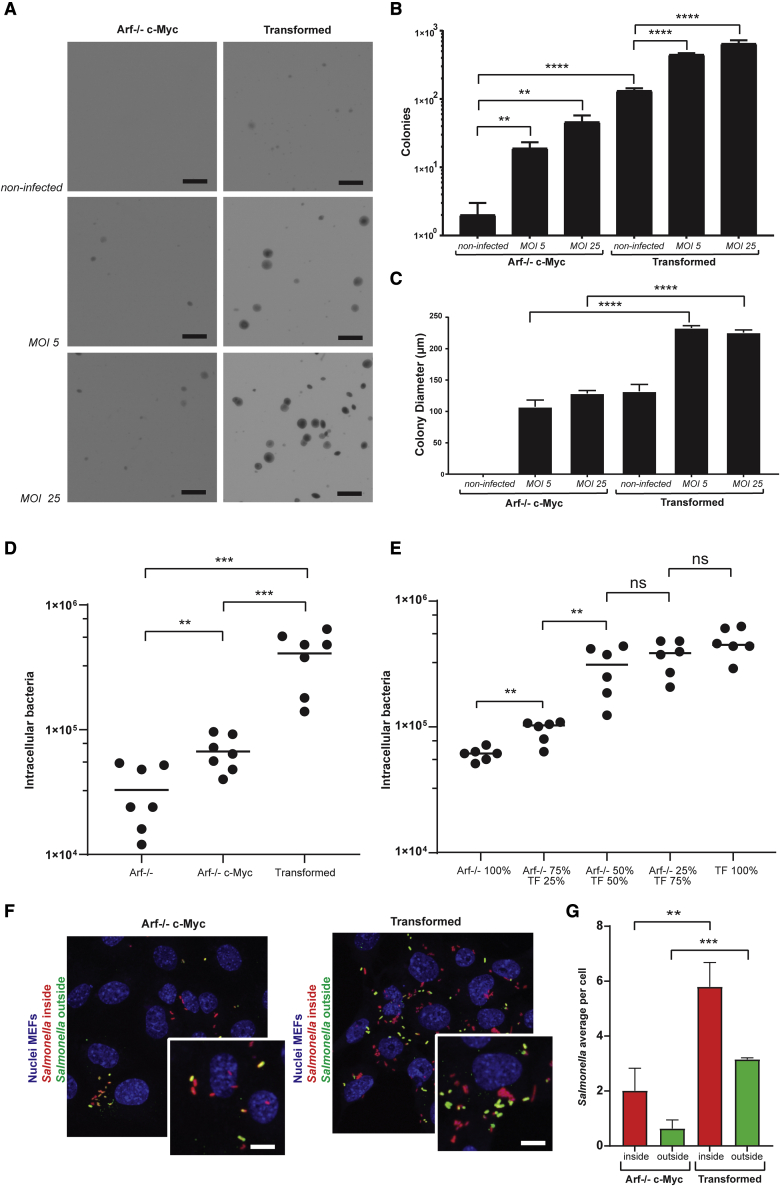
NTS preferentially infects (pre-)transformed cells, and repetitive NTS infections increase cellular transformation *in vitro* (A) Representative images of anchorage-independent growth assay of *Arf*-deficient MEFs overexpressing c-Myc that have not been previously exposed to *NTS* (top rows: *Arf*^−/−^ c-Myc) and transformed *Arf*-deficient MEFs overexpressing c-Myc that have been previously exposed to *NTS* (bottom rows: transformed). *Arf*^−/−^ c-Myc or transformed MEFs either non-infected, infected with an MOI of 5, or infected with an MOI of 25 are indicated in the left, middle, and right column, respectively. Scale bar, 750 μm. (B) Average number of soft-agar colonies per well of *Arf*^−/−^ c-Myc and transformed MEFs overexpressing c-MYC that have been either non-infected, infected with an MOI of 5, or infected with an MOI of 25. The data are expressed as mean ± SD; one-way ANOVA, ∗∗p < 0.01, ∗∗∗∗p < 0.0001. (C) Average colony diameter of anchorage-independent growth of naive and transformed *Arf*-deficient MEFs overexpressing c-MYC that have been either non-infected, infected with an MOI of 5, or infected with an MOI of 25. The data are expressed as mean ± SD; one-way ANOVA, ∗∗∗∗p < 0.0001 (D) CFU counts of intracellular bacteria in *Arf*^−/−^, *Arf*^−/−^ c-Myc, and transformed MEFs after infection with NTS at MOI 25 for 1 h. The data are expressed as mean ± SD; one-way ANOVA, ∗∗p < 0.01, ∗∗∗p < 0.001. (E) CFU counts of intracellular bacteria in mixed populations of *Arf*^−/−^ and transformed MEFs after infection with NTS at MOI 25 for 1 h. The data are expressed as mean ± SD; one-way ANOVA, ∗∗p < 0.01. (F) Representative images of intra- (inside) and extracellular (outside) NTS bacteria in *Arf*^−/−^ c-Myc and transformed MEFs after infection with NTS at MOI 25 for 1 h. Scale bar, 10 μm. (G) Quantification of intra- (inside) and extracellular (outside) NTS bacteria in *Arf*^−/−^ c-Myc and transformed MEFs after infection with NTS at MOI 25 for 1 h. The data are expressed as mean ± SD; one-way ANOVA, ∗∗p < 0.01, ∗∗∗p < 0.001. Results in this figure derive from three independent experiments with technical triplicates.

We have established a minimal tissue culture model for monitoring *Salmonella*-induced transformation. This model includes mouse embryonic fibroblasts (MEFs) engineered to mimic two steps toward transformation: *Arf* deficiency (resulting in TP53 inactivation) and overexpression of c-Myc (named *Arf*^−/−^ + c-Myc). Both TP53 mutations and c-Myc overexpression were also observed in gallbladder carcinoma from patients with a history of *S*. Typhi infection.^6^ To test whether repeated exposures to NTS increased the rate of transformation, *Arf*^−/−^ + c-Myc MEFs were firstly infected with *S*. Typhi (MOIs 5 and 25) and seeded in soft agar. As previously reported,^6^ the acquired capacity of the cells to grow and form colonies—in an anchorage-independent manner—which is an established hallmark of transformed cells, resulted from NTS-induced transformation (Figures 3A and 3B; *Arf*^−/−^ + c-Myc, MOIs 5 and 25). Of note, this phenotypic change of cell behavior may be directly or indirectly linked to (epi)genetic modifications.^6^ As control, non-infected *Arf*^−/−^ + c-Myc MEFs were included that failed to induce colony formation (Figures 3A and 3B; *Arf*^−/−^ + c-Myc, non-infected). Several colonies of NTS-infected *Arf*^−/−^ + c-Myc MEFs were then isolated from soft agar and cultured under normal 2D cell culture conditions. Throughout culturing, no remaining NTS was observed in these cells, as reported previously.^6^ These procedures resulted in the establishment of *Arf*^−/−^ + c-Myc MEF cell lines with a history of NTS infection (hereafter referred to as transformed MEFs). Following re-seeding of these NTS-transformed cells, cells remained able to form colonies in soft agar, as reported previously^6^ (Figures 3A and 3B). To evaluate the effect of repeated NTS exposures on cell transformation, transformed MEFs were re-infected prior to re-seeding in soft agar, which yielded significantly more colonies (Figures 3A and 3B, transformed**,** comparing non-infected with MOIs 5 and 25). This increase was NTS-dose dependent, as an MOI of 25 resulted in significantly more colonies than an MOI of 5 (Figures 3A and 3B, transformed, comparing MOI 5 with MOI 25). Remarkably, colonies of the transformed cells were also larger following a re-infection, indicating that these colonies proliferated faster than the reseeded *Arf*^−/−^ + c-Myc MEFs or non-infected transformed MEFs (Figure 3C). Increased transformation upon repeated infections was consistent among various subsets of the NTS-transformed *Arf*^−/−^ + c-Myc MEF isolates (isolated colonies) (Figure S4). Moreover, the accumulative transformative effect of a successive infection persisted after two successive rounds of repeated infection, whereby a second infection of transformed MEFs again resulted in significantly more colonies than their non-infected controls (Figure S5). An initial and repetitive infection with a NTS T3SS mutant ΔprgH significantly reduced the transformative effect of an NTS infection, indicating that a functional SPI1 T3SS is involved during both initial and recurrent NTS-infection-induced transformations (Figures S6D and S6E).

### NTS preferably infects (pre-)transformed cells *in vitro*

As observed in our mouse cohort, tumor tissues were significantly more colonized by NTS compared with adjacent non-tumor tissues. In line with these observations, it has been reported that NTS preferentially accumulates in tumors when compared with other organs a week after systemic injection.^27^ Moreover, specific targeting of host cells by NTS has been reported for particular morphological and microenvironmental features.^28^ To evaluate whether NTS specifically targets (pre-)transformed host cells, we compared the efficiency of NTS infection of MEF cell lines that harbored either one (*Arf*^−/−^) or two pre-transforming mutations (*Arf*^−/−^ and c-Myc expression) to the NTS infection efficiency of transformed MEFs. Intracellular bacterial counts of transformed MEFs were found to be significantly higher than the intracellular bacterial counts of the pre-transformed *Arf*^−/−^ and *Arf*^−/−^ + c-Myc MEFs, indicating that transformed MEFs are more susceptible to NTS invasion than pre-transformed MEFs (Figure 3D). Moreover, intracellular bacterial counts of *Arf*^−/−^ + c-Myc MEFs were significantly higher than the intracellular bacterial counts of *Arf*^−/−^ MEFs, also correlating infection efficiency to the transformation state (Figure 3D). The selectivity of NTS for infecting transformed MEFs was further confirmed in a mixed culture of pre-transformed *Arf*^−/−^ MEFs and transformed MEFs. Increasing the proportion of transformed MEFs within the overall cell population correlated with a similar increase in total intracellular NTS numbers, further illustrating that transformed MEFs are infected by NTS with higher efficiency (Figure 3E). Fluorescence microscopy of NTS infected *Arf*^−/−^ c-Myc MEFs and transformed MEFs confirmed higher numbers of Salmonellae in transformed MEFs compared with pre-transformed MEFs. To distinguish intracellular NTS from cell-surface-bound NTS (i.e., not eliminated during washing steps), we used an NTS strain constitutively expressing a dsRed fluorophore and immunolabeled the extracellular salmonellae. Of note, NTS counts at the cell surface of transformed MEFs were also higher compared with pre-transformed MEFs, suggesting that the transformed state increased the host cell adherence of NTS (Figures 3F and 3G). Infection of the transformed MEFs with a NTS T3SS mutant ΔprgH abolished the observed increased host cell adherence and increased host cell infection, indicating that a functional SPI1 T3SS remains required to infect the transformed MEFs (Figures S6A–S6C). Together, these *in vitro* data demonstrate a privileged tropism of NTS for cells with the highest level of transformation.

## Discussion

Colonic microbiota contribute to both the development and progression of CC,^29^^,^^30^^,^^31^ and the rising incidence of CC in the younger population in the Western world, for partly unknown reasons, stresses the need to thoroughly investigate their contribution (also in relation to external factors like diet and physical activity) to CC development. Research into the contribution of bacterial infections to CC development has the potential to unravel novel, complex biological mechanisms behind colon carcinogenesis, as well as facilitate early cancer detection and identify targets for personalized therapy. Understanding the prognostic value of *Salmonella* infection may provide a basis for the development of guidelines and criteria advocating certain screenings and follow ups in groups of people particularly exposed to this pathogen.^32^

NTS infections can induce oncogenic transformation of pre-transformed cells upon targeting and manipulation of essential signaling pathways.^6^ In long-lasting and severe NTS infections, NTS is more likely to encounter a pre-transformed colon cell, increasing the risk of oncogenic transformation. This has already been suggested by the epidemiological association between severe NTS infections and increased CC risk.^7^ It then follows that the sum of multiple NTS infections, which people are known to acquire throughout life,^16^^,^^17^^,^^18^ are similarly conceivable to induce CC.

To test this hypothesis, we assessed the risk of CC development as a function of the magnitude of NTS exposure, as determined by serology, by combining data from different national representative registries in a retrospective cohort study. This provided insights into the frequency of NTS infection and revealed an increased risk of developing CC among people with increased NTS seroincidence before 60 years of age, before other factors related to advanced age become a dominant risk factor for CC.^7^ The magnitude of exposure to NTS was found to be significantly associated with CC risk among people with a high SES. A higher SES is often associated with a more sedentary occupation (i.e., the so-called “white collar” professions), which is a known risk factor for CC.^33^ Hence, it seems that colon carcinogenesis fueled by increased exposure to NTS interacts with other drivers of CC, specifically depending on the presence or absence of other risk factors for CC like age and lifestyle (which SES is a proxy for), an effect of NTS infection on CC.

This seroepidemiological study demonstrated that an increased rate of *Salmonella* infection should be considered an environmental risk factor for CC. Epidemiology by definition provides, however, only a correlation between the frequency of mild *Salmonella* infections and CC risk. Moreover, we measured the frequency of infection prior to later CC diagnosis. While we have no information about the rate of infections between serum acquisition and CC diagnosis, we assumed that this did not significantly alter. In addition, the time to tumor progression can be years, and we have no information about the time when the first colon cells became definitively transformed. While we can exclude factors like SES and gender, we cannot exclude other factors (such as co-infections with other pathogens, food, and others) that may also contribute to transformation. While we analyzed a dataset that allows us to test the correlation between rate of mild infections (as detected by the immune system rather than the person) and CC, an independent confirmation would strengthen the case. To move from correlation to causality, experimental validation is critical, as provided in this study.

Previously, high doses of NTS were shown to contribute to CC formation in pre-transformed mouse models.^6^^,^^24^ Here, we observed that multiple low doses of NTS accelerate *in vivo* tumor formation in a manner similar to a single high dose, thereby indicating that repetitive low-dose exposures to NTS trigger tissue and tumor proliferation in a comparable manner to a single high-dose NTS exposure. Moreover, after both repetitive low-dose infections and a single high-dose infection, NTS similarly colonized colon tissues of the mice at the end of the study as deduced by pathology. This could be attributed to our observation that (pre)-transformed cells are more efficiently infected by NTS, whereby a repetitive low inoculum could suffice to target and colonize (pre-)transformed tissues with similar efficiency as a high inoculum. NTS persistence at the end of the *in vivo* study suggests acceptance of the infection without signs of inflammation as deduced from the observations that no significant differences in serum cytokines and chemokines were observed between the groups at that point (Figure S7). This is in line with previous studies in which the NTS infection induces high immune cytokines in the blood of the mice after 1 week but then drops to normal levels at 10 weeks post infection.^34^ These outcomes furthermore demonstrate, together with the outcomes of our simple cell culture system experiments (Figure 3), our sporadic CC mouse model (Figure S3) and our previous findings, that NTS-induced CC formation does not appear to involve major immunomodulatory mechanisms but instead relies on the activation of the host’s β-catenin, MAPK, and AKT signaling pathways by the *Salmonella* effectors AvrA, SopE, SopE2, and SopB.^6^^,^^9^^,^^10^^,^^11^^,^^24^

The higher NTS seroincidence among prospective CC patients and the oncogenic role of recurrent low-dose NTS infections observed in tissue culture and mouse models identify recurrent low-dose NTS infections as a cumulative risk factor for CC development. Low-dose NTS infections can be easily obtained from many sources. Indeed, numerous NTS serovars colonize animals and environmental reservoirs, with *S*. Enteritidis transmission being essentially foodborne, whereas *S*. Typhi is more ubiquitous.^33^ While exposure via food can, in principle, be prevented, elimination of environmental exposure to NTS is practically impossible. Like sunlight, mild and recurrent NTS infections may represent a hitherto unknown environmental risk factor for CC that cannot be avoided, and this may be the case for other cancers and bacterial species as well.^35^

### Limitations of the study

The study determines the rate of mild infection by *Salmonella* in the past and couples that to CC diagnosis. These mild infections may not be noted by people but are by their immune system, which detects such an infection once every few years. The IgG, IgM, and IgA titers against *Salmonella* lipopolysaccharide (LPS) in the sera of people that later developed CC versus an age- and gender-matched control group indicated the exposure at the time of serum sampling. CC is diagnosed years later, and we do not have data between serum sampling and CC diagnosis. The assumption is that *Salmonella* exposure is not significantly altered across time. We do not know the time between the *Salmonella* infection of a pre-transformed colon cell and CC diagnosis, but this is likely many years.

The mouse and tissue culture experiments show that pre-transformed cells are more easily infected and that *Salmonella* then accelerates tumor growth. Since *Salmonella* can transform pre-transformed colon cells, the *Salmonella* bacteria have to meet this cell. Pre-transformed colon cells are more frequent in the elderly population, but CC may then also spontaneously develop. The chance of a *Salmonella* bacterium meeting a pre-transformed colon cell is higher, in the case of older people, following a serious infection by *Salmonella* requiring hospitalization and—obviously—following a higher frequency of mild infections by this bacterium, which is widely present in the environment.

In this study, we used a well-established cell transformation model with fibroblasts forming colonies in soft agar to estimate the anchorage-independent growth ability of cells. This old model for cancer formation allows us to measure changes of cellular behavior but does not provide direct follow up of molecular changes. The molecular mechanism of cell transformation following *Salmonella* infection is not currently fully understood, which is why the direct measurement of genetic or epigenetic modifications is not performed. The finding that repetitive infection accelerates cell transformation (i.e., their ability to form colonies in soft agar) will foster the investigation of the molecular mechanism behind this transformation in future studies.

## STAR★Methods

### Key resources table

**Table:** 

REAGENT or RESOURCE	SOURCE	IDENTIFIER
**Antibodies**		

Anti-BrdU antibody	Abcam, Cambridge	Cat. #: ab1893; RRID:AB_302659
*Salmonella* Typhimurium 0–4 antibody	Santa Cruz, Dallas, TX, USA	Cat. #: sc-52224; RRID:AB_630225
rabbit polyclonal anti-*S*.Typhimurium LPS	Difco, Detroit	N/A

**Bacterial and virus strains**		

*Salmonella* Typhimurium 1090200009	RIVM – clinical strain	1090200009
*Salmonella* Enteritidis 1090301578	RIVM – clinical strain	1090301578
*Salmonella* Enteritidis 1091100412	RIVM – clinical strain	1091100412
*Salmonella* Typhimurium 1090601671	RIVM – clinical strain	1090601671
*Salmonella* Typhimurium 1090404321	RIVM – clinical strain	1090404321
*Salmonella* Enteritidis 1091302626	RIVM – clinical strain	1091302626
*Salmonella* Typhimurium SL1344	ATCC	ATCC SL1344

**Chemicals, peptides, and recombinant proteins**		

Hematoxylin&Eosin	Leica Biosystems Inc., Buffalo Grove, IL, USA	Cat. #: 3801571 / 3801606
Lipopolysaccharides *S*. Enteritidis (O-antigens 1,9,12)	SIGMA, Copenhagen	Cat. #: L7770
Lipopolysaccharides *S*. Typhimurium (O-antigens 4,5,12)	SIGMA, Copenhagen	Cat. #: L6143
Sodium pentobarbital	Sigma-Aldrich, St. Louis, MO, USA	Cat. #: P5178
Buffered Peptone Water	Sigma-Aldrich, St. Louis, MO, USA	Cat. #: 77187
Azoxymethane (AOM)	Sigma-Aldrich, St. Louis, MO, USA	Cat. #: A5486
Dextran sodium sulphate (DSS)	MP Biomedicals, LLC., Solon, Ohio, USA	Cat. #: 160110
gentamicin	Gibco	Cat. #: 15710064
low melting point agarose	UltraPure™, Invitrogen	Cat. #: 16520050
DAPI Nucleic Acid Stain	Life Technologies, Carlsbad, CA, USA	Cat #: D1306

**Critical commercial assays**		

ProcartaPlex Mo Cytok./Chemok. Convenience Panel 1 26plex	Thermo Fisher Scientific, Waltham, MA, USA	Cat. number: EPXR260-26088-901

**Experimental models: Cell lines**		

Mouse Embryonic Fibroblasts (MEFs) were derived from *Arf*-deficient C57BL/6 mice	PMID: 26028364	N/A

**Experimental models: Organisms/strains**		

Mouse: C57BL/6J	Jackson Laboratory (Bar Harbor, ME, USA)	Strain #:**000664**
Mouse: APC15loxp: B6.129P2-*Apc*^*tm1Rsmi*^/RfoJ	Jackson Laboratory (Bar Harbor, ME, USA)	Strain #:**029275**
Mouse: CDX2P-NLS Cre: B6.Cg-Tg(CDX2-cre)101Erf/J Common Name: CDX2P-NLS Cre	Jackson Laboratory (Bar Harbor, ME, USA)	Strain #:**009350**

**Recombinant DNA**		

pLZRS-GFP(ires)-HA	PMID: 26028364	N/A
pGG2 plasmid coding dsRed	PMID: 19800337	N/A

**Software and algorithms**		

GraphPad Prism 5	GraphPad Software	N/A

### Resource availability

#### Lead contact

Further information and requests for resources and reagents should be directed to and will be fulfilled by Jacques Neefjes (j.j.c.neefjes@lumc.nl).

#### Materials availability

All unique/stable reagents generated in this study are available from the lead contact, Jacques Neefjes (j.j.c.neefjes@lumc.nl) with a completed Material Transfer Agreement.

### Experimental model and subject details

#### Seroepidemiological study design

A retrospective matched cohort study was performed based on two linked data sets. The first data set derived from a nationwide cross-sectional serological survey conducted in the Netherlands in October 1995-December 1996, the ‘PIENTER-1’ study.^18^ The design and rationale of PIENTER-1 are described in detail elsewhere.^18^ In brief, a two-stage cluster sampling design, with 48 municipalities nested in five study-defined regions and age-stratified random sampling applied within these municipalities, was performed. In total, 18,217 people were invited to complete an epidemiological questionnaire and to donate a blood sample. Informed consent was obtained for all participants. Data on the neighbourhood socio-economic status (SES: classified as low, intermediate, and high, based on a standardized index including income, occupation, and education) per postal code area was obtained from Statistics Netherlands (www.cbs.nl). In total, 9948 persons provided a serum sample. The second data set, maintained by the Dutch Association of Comprehensive Cancer Centres (IKNL) (www.iknl.nl), was derived from the Netherlands Cancer Registry (NCR). This registry covers all residents in the Netherlands, the data are more than 95% complete, and includes data on patients diagnosed with CC (ICD-O-3 codes: C180-C189) since 1990. These data also include the colon subsite (proximal, distal) in which the tumour has been found.

Statistics Netherlands (CBS) acted as a trusted third party for data anonymization and linkage by adding a Record Identification Number (RIN) as unique identifier for each individual in the two data sets. Birth date, gender, residence location, and date of registration formed the basis for the derivation of the RIN numbers. To this end, CBS used a reference database containing all mutations due to death or relocation in the Dutch population, including a complete housing history of all Dutch residents. After the RIN numbers were added, all personal identifiers were removed. Based on RIN numbers, the participants of the PIENTER-1 study were linked to the NCR data on patients with diagnosed CC.

All data sets were cleared from duplicates. CC patients with a date of diagnosis falling after the end of the study period (December 31^st^, 2017) were censored. As a previous study highlighted a significantly increased risk of cancer only in the proximal part of the colon after reported NTS infection,^7^ we excluded cases with cancer in the distal part of the colon. After linking the PIENTER-1 records to those of the CC patients in the NCR data set, 36 matches were found, i.e. 36 participants in PIENTER-1 who were diagnosed with cancer in the proximal colon in the period between January 1^st^, 1998 and December 31^st^, 2017 (end of the present study period). Each of these 36 CC patients was matched at 1:2 ratio to other PIENTER-1 participants who were not diagnosed with any CC and did not die during the study period. Matching was based on age (±1 year), gender, self-reported educational level (low = primary, lower vocational or lower secondary education; intermediate = intermediate vocational, intermediate secondary or higher secondary education; high = higher vocational and university education), and smoking behavior (smoker, no-smoker, unknown), as reported in the PIENTER-1 study.

Median follow-up time (i.e. time between entry into the cohort and CC diagnosis for the cases or censoring for the matched controls) was 13 years (mean 12 years, IQR 6–16 years), amounting to 1293 person-years at risk in total. The median age at exit from the cohort (i.e. CC diagnosis for the cases or censoring for the matched controls) was 75 years (mean 72 years, IQR 65–80 years). The cohort was mainly composed by persons with a low to intermediate educational level and living predominantly in neighborhoods of low socio-economic status (SES) (Table S1).

#### NTS-infected CC mouse model

Mouse experiments were performed using a specific pathogen–free male and female C57BL/6 AOM + DSS CC model, that has been extensively used as a model system to investigate the accelerating effect of NTS infections to the multi-step CC formation process.^24^ Animal experiments were performed with 50 male and 50 female C57BL/6 mice aging 6–7 weeks old (The Jackson Laboratory, Bar Harbor, ME, USA).^9^^,^^24^ The colon-specific APC knockout (APCΔCEC) mice were generated by breeding between APC15loxp mice (Jackson Laboratory, 029275) and CDX2P-NLS Cre mice (Jackson Laboratory, 009350). The 6–8 week old female and male APC15loxp and APCΔCEC mice were used for experiments. All animal work was approved by University of Illinois at Chicago Committee on Animal Resources (AAC 18–216). Euthanasia was accomplished via sodium pentobarbital (100 mg per kg body weight) I.P., followed by cervical dislocation. All methods were carried out in accordance with the approved guidelines by the Committee on Animal Resources.

#### Bacterial strains for animal model and growth condition

Six clinical isolates, including *Salmonella* Typhimurium 1090200009, *Salmonella* Enteritidis 1090301578, *Salmonella* Enteritidis 1091100412, *Salmonella* Typhimurium 1090601671, *Salmonella* Typhimurium 1090404321, and *Salmonella* Enteritidis 1091302626, were used for the morbidity and mortality animal studies. The clinical isolate *Salmonella* Typhimurium 1090404321 was used for the long-term colon cancer mouse model study.

#### Bacterial strains and cell lines for *in vitro* experiments

*S*. Typhimurium strain SL1344 was a courtesy of S. Méresse and PrgH-deficient *S*. Typhimurium SL14028 was provided by D. Holden.^36^ Mouse Embryonic Fibroblasts (MEFs) were derived from *Arf*-deficient C57BL/6 mice. MEFs overexpressing c-Myc were generated by retroviral transduction using a pLZRS-GFP(ires)-HA backbone. MEFs were cultured at 37°C, 5% CO_2_ in Dulbecco′s Modified Eagle′s Medium (DMEM) (Invitrogen).^6^

### Method details

#### Serological analyses and seroincidence calculation

The serum samples of the 36 CC cases and the 72 persons without CC (i.e. ‘controls’) were retrieved from the PIENTER-1 serum bank and tested for anti-*Salmonella* IgA, IgM, and IgG concentrations using a validated mixed ELISA based on commercially available lipopolysaccharides (SIGMA, Copenhagen) of the two most common serovars, namely Enteritidis (O-antigens 1,9,12) and Typhimurium (O-antigens 4,5,12), as capture antigens in solid phase. A detailed description of this ELISA and its validation has been published previously.^21^ For each sample, the concentrations of each Ig isotype were measured separately and expressed as optical density (OD) units. These OD values were then used to estimate, for each sample, the seroincidence of NTS infection, i.e. the average number of NTS infections per person-year as a measure of NTS infection pressure (or force of infection) in the person in question. This was done using the Bayesian ‘back-calculation’ model provided for in the R package called ‘seroincidence’, which has been described in detail elsewhere^16^^,^^17^^,^^20^ and has been adopted as the standard seroincidence calculator by the European Centre for Disease Control (ECDC) (https://ecdc.europa.eu/en/publications-data/seroincidence-calculator-tool). In brief, the model is based on the kinetics of IgG, IgM, and IgA observed during an 18-month follow-up study with repeated bleeding of 302 Danish adult patients with stool culture-confirmed NTS infections. The model used these data as reference values for peak levels and decay rates of IgG, IgM, and IgA concentrations over time after *Salmonella* infection so that the Ig values measured in a sample can be modelled as a function of time since last seroconversion, taking into account inter-individual variation, thereby estimating an annual seroincidence for any observed set of Ig values in a single sample. This model has been used in several studies on immuno-dynamics of NTS^17^^,^^20^ and has been adapted to *Campylobacter*^37^^,^^38^^,^^39^^,^^40^ and *Yersinia enterocolitica*^41^ as well.

#### NTS-infected CC mouse model

After setting-down for one week in the animal facility, the mice were infected with either a) a single high dose of 1 × 10^4^ CFU (100-μL suspension in HBSS) S. Typhimurium, b) repetitive low doses of 1 × 10^1^ CFU (100-μL suspension in HBSS) *S*. Typhimurium or c) treated with sterile HBSS (control) by oral gavage, as previously described.^24^ Non-agitated microaerophilic bacterial cultures were prepared by inoculating 0.01 mL of a stationary-phase culture to 10 mL Buffered Peptone Water (Sigma-Aldrich, St. Louis, MO, USA) followed by overnight incubation (∼18 h) at 37°C. After NTS gavage, the carcinogen AOM was administrated through intraperitoneal injection with the dose based on body weight (10 mg/kg).^24^ After a 7-day recovery period, the inflammatory agent dextran sodium sulphate (DSS) was administrated at 2% in drinking water for seven days. This DSS treatment was repeated at 5 and 9 weeks. In the group of repetitive low dose infections, oral gavage with 1 × 10^1^ CFU (100-μL suspension in HBSS) of *S*. Typhimurium was repeated at 4 and 8 weeks. Throughout the experiment, mice were weighed and monitored regularly. At 16 weeks post NTS infection, tumors and tissue samples were collected. Tumor counts and measurements were performed in a blinded fashion under a stereo-dissecting microscope (Nikon SMZ1000, Melville, NY, USA). The tumor volume (V) was calculated with caliper measurements using formulas V= (W^2^ × L)/2 as described before.^42^

#### *In vitro* NTS infection, CFU, microscopy and anchorage-independent growth assays

NTS infection of MEFs cells was performed as described previously.^10^ In brief, *S*. Typhimurium strain SL1344 was grown overnight at 37°C in LB medium, supplemented with 100 μg/mL ampicillin throughout the bacterial culturing. The next day, the bacteria were sub-cultured at a dilution of 1:33 in fresh LB medium and incubated for 2 h at 37°C while shaking. Cells were infected with NTS at the indicated MOI in DMEM medium without antibiotics for 20 min at 37°C, 5% CO_2_ in a tissue culture chamber and then incubated in the presence of 100 μg/mL gentamicin (GIBCO) for 1 h to eliminate extracellular bacteria. In case of CFU or microscopy experiments cells were then lysed and plated on LB plates or fixed with 4% PFA for 10 min at room temperature, respectively. In case of anchorage-independent growth assays MEFs were cultured for another 2 h in the presence of 10 μg/mL gentamicin. The infected MEFs were subsequently collected and resuspended in DMEM medium supplemented with 10 μg/mL gentamicin and 0.35% low melting point agarose (UltraPure™, Invitrogen) and were poured on a soft agar bottom layer consisting of 0.7% low melting point agarose in DMEM with 10 μg/mL gentamicin. Anchorage-independent cell growth and number of soft agar colonies were assessed after 1–3 weeks of incubation at 37°C, 5% CO_2_ using GelCountTM (Oxford Optronix, UK). For microscopy analysis *Salmonella* bacteria were transformed with a GFP or dsRed plasmid prior to the experiment and fixed slides were stained with rabbit polyclonal anti-*S*. Typhimurium LPS (Difco, Detroit, MI) and DAPI (Life Technologies). Images were acquired using a Leica TCS SP8 (Leica Microsystems, Wetzlar, Germany) at 40x or 63x magnification.

#### Histological testing

Tissues were fixed in 10% neutral buffered formaldehyde for 4–12 h, then transferred into 70% ethanol and processed by standard techniques. Sections (4μm) were stained with hematoxylin and eosin.

#### Immunohistochemistry

Tissues were fixed in 10% neutral-buffered formaldehyde for overnight, then transferred into 70% ethanol the next day and processed by standard techniques. Immunohistochemistry staining of target protein was performed on paraffin-embedded sections (4 μm). Briefly, the paraffin sections were baked in an oven at 56°C for 30 min. The sides were deparaffinized and rehydrated in xylene, followed by graded ethanol washes at room temperature. Antigen retrieval was achieved by boiling the slides in a microwave oven with 0.01 M, pH 6.0 sodium citrate buffer. Then, the slides were incubated in hydrogen peroxide (3% H_2_O_2_ in PBS) for 10 min, followed by incubation in 5% fetal bovine serum/PBS for 1 h at room temperature. Purified Anti-BrdU antibody (Abcam, Cambridge, MA, USA) was used in this study.^43^

#### Immunofluorescence and confocal imaging

Fresh tumors were fixed in 10% neutral buffered formalin followed by paraffin embedding. For immunofluorescence staining, slides were incubated in 5% bovine serum albumin (BSA) with 0.1% goat serum in PBS for 1 h at room temperature to reduce nonspecific background. The samples were incubated overnight at 4°C with primary antibody at 1:100 dilution. The sections were then incubated with secondary antibodies and DAPI for 1 h at room temperature, and they were examined with confocal microscope as described before.^43^^,^^44^ The mouse monoclonal antibody for *S*. Typhimurium 0–4 (Santa Cruz, Dallas, TX, USA) was used in this study.

#### Luminex immunoassays

The cytokines and chemokines in the plasma samples from the studied animals were assessed using the ProcartaPlex Mouse Cytokine/Chemokine Convenience Panel 1 26plex (Thermo Fisher Scientific, Waltham, MA, USA) according to the manufacturer’s instruction. Briefly, after adding magnetic beads, 25μL of plasma samples were added and followed by detection of antibody and streptavidin-PE provided by the kit. The plate was read on a MAGPIXTM system platform (Millipore Sigma, Burlington, MA, USA) after adding reading buffer.

### Quantification and statistical analysis

#### Statistical analysis of seroepidemiological data

The goal of the analysis was to assess whether NTS seroincidence was a significant predictor of CC. Cox proportional hazards models with attained age as the time scale were used to calculate hazard ratios (HR) and 95% confidence intervals (95%CI) for CC (failure event) as a function of the NTS seroincidence (continuous predictor variable). Follow-up started at cohort entry (i.e. serum sampling at the PIENTER-1 study) and ended at CC diagnosis for both the cases and their matched controls (censoring). As the follow-up time was equal for the members of each matched set, the Breslow method for ties in follow-up time produced HRs that corresponded to risk ratios.^45^ A clustered sandwich estimator for variance was used to account for the matched sets, which shown to yield robust estimates of variance for hypothesis testing^46^ and generally produce results comparable to frailty models.^47^

Stratified analyses were performed according to age at sampling (defined as <60 *vs*. ≥60 years, as this was the mean age in our sample and a previous study showed that the potential effect of NTS infection on CC development is unlikely to be observed after that age given the prominent role of other risk factors that may ‘dilute’ the effect of the infection),^7^ as well as gender, neighbourhood SES, educational level, and smoking status, to assess whether there were modifications of the effect of NTS infection pressure on CC risk according to these strata. The two-way interactions between seroincidence and the aforementioned variables were assessed in separate models adjusted for the other variables. However, to avoid collinearity due to the strong association between educational level and neighbourhood SES, only one of these two factors was included as covariate based on the best model fit as revealed by the Akaike's information criterion. Proportional hazard assumptions were verified using graphical and residual-based methods and found to be met. Statistical analysis was performed using STATA 15 (StataCorp, LP, College Station, Texas, USA).

#### Statistical analysis of mouse experiments

For the mouse model related experiments, data were expressed as mean ± SD. One-way ANOVA was performed to the statistical analysis in the animal studies. All statistical tests were two-sided, and p-values <0.05 were considered statistically significant. The statistical analyses of experimental data were performed with GraphPad Prism 5.

#### Statistical analysis of *in vitro* NTS infection

Experiments were performed independently 3 times and included technical triplicate. The data were represented in figures as mean ± SD. Statistical analysis were perform with one-way ANOVA, p-values were noted as follow: ∗∗p < 0.01, ∗∗∗p < 0.001.

## Data Availability

• All data reported in this paper will be shared by the lead contact upon request. • This study did not generate new sequencing data or code. • Any additional information required to reanalyze the data reported in this paper is available from the lead contact upon request.

## Supplemental information

Document S1. Tables S1 and S2 and Figures S1–S7

Document S2. Article plus supplemental information
