# Supplementary material for: Repetitive non-typhoidal Salmonella exposure is an environmental risk factor for colon cancer and tumor growth
Source: Cell Rep Med. 2022 Dec 20;3(12):100852. doi: 10.1016/j.xcrm.2022.100852 (PMC9798023; doi:10.1016/j.xcrm.2022.100852)
Supplement: Document S1. Tables S1 and S2 and Figures S1–S7 [file mmc1.pdf]

Cell Reports Medicine, Volume *3*

Supplemental information

Repetitive non-typhoidal *Salmonella* exposure is an environmental risk factor for colon cancer and tumor growth

Daphne M. van Elsland, Janneke W. Duijster, Jilei Zhang, Virginie Stévenin, Yongguo Zhang, Lang Zha, Yinglin Xia, Eelco Franz, Jun Sun, Lapo Mughini-Gras, and Jacques Neefjes

Supplementary Tables

Supplementary Table 1. **Baseline characteristics of the study cohorts. Related to Figure 1.**

	Individuals who developed colon cancer during the follow-up period	Individuals who did not develop colon cancer during the follow-up period*
Gender		
Male	15 (42%)	30 (42%)
Female	21 (58%)	42 (58%)
Age at entry		
<60 years	15 (42%)	30 (42%)
≥60 years	21 (58%)	42 (58%)
Educational level at entry**		
Low	14 (39%)	28 (39%)
Intermediate	14 (39%)	28 (39%)
High	8 (22%)	16 (22%)
Neighbourhood SES at entry***		
Low	24 (67%)	49 (68%)
Intermediate	5 (14%)	10 (14%)
High	7 (19%)	13 (18%)
Smoking at entry		
Smoker	6 (17%)	12 (17%)
Non-smoker	29 (80%)	58 (80%)
Unknown	1 (3%)	2 (3%)
Follow-up time		
<5 years	6 (16%)	12 (16%)
5-15 years	15 (42%)	30 (42%)
>5 years	15 (42%)	30 (42%)

*Matched to the colon cancer patients at a 1:2 ratio based on gender, age at entry (±1 year), educational level and smoking status. **Low=primary, lower vocational or lower secondary education; intermediate=intermediate vocational intermediate secondary or higher secondary education; high=higher vocational and university education. ***Socio-economic status, classified as low, intermediate and high based on a standard index including income, occupation and education per postal code area ('neighbourhood') obtained from Statistics Netherlands (www.cbs.nl).

Supplementary Table 2: *Salmonella* sero-incidence rates and colon cancer risk by gender, age, ethnicity, educational level, socioeconomic status and smoking. Related to Figure 1.

	Mean (95%CI) *Salmonella* sero-incidence among colon cancer cases	Mean (95%CI) *Salmonella* sero-incidence among controls	Person-years at risk	Hazard ratio (95%CI)	P-value
Overall	0.94 (0.55-1.32)	0.73 (0.57-0.88)	1293	1.24 (0.82-1.88)§	0.302
Age at entry					
**<60 years**	**1.26 (0.48-2.04)**	**0.96 (0.64-1.28)**	**617**	**1.41 (1.03-1.94)†**	**0.033**
≥60 years	0.71 (0.40-1.01)	0.58 (0.48-0.67)	676	0.77 (0.29-2.01)†	0.704
Gender					
Male	1.13 (0.32-1.94)	0.73 (0.61-0.85)	581	1.46 (0.97-2.22)§	0.072
Female	0.80 (0.50-1.10)	0.73 (0.47-0.99)	712	1.06 (0.68-1.64)§	0.810
Educational level at entry*					
Low	0.55 (0.38-0.73)	0.54 (0.42-0.65)	424	0.83 (0.23-2.94)§	0.771
Intermediate	0.67 (0.47-0.88)	0.90 (0.55-1.25)	564	1.01 (0.59-1.73)§	0.965
High	2.07 (0.71-3.44)	0.78 (0.60-0.96)	305	1.26 (0.96-1.66)§	0.102
Neighbourhood SES at entry**					
Low	0.63 (0.47-0.78)	0.73 (0.51-0.95)	827	0.87 (0.47-1.60)†	0.656
Intermediate	0.92 (0.22-1.62)	0.57 (0.44-0.70)	202	1.03 (0.71-1.49)†	0.866
**High**	**2.01 (0.44-3.58)**	**0.85 (0.65-1.08)**	**264**	**1.32 (1.03-1.69)†**	**0.027**
Smoking at entry					
Smoker	1.33 (0.00-3.11)	0.65 (0.36-0.93)	242	1.46 (0.97-2.19)†	0.068
Non-smoker	0.88 (0.58-1.17)	0.76 (0.57-0.94)	988	1.06 (0.73-1.52)†	0.234
Unknown	0.32 (0.32-0.32)	0.48 (0.00-1.27)	63	-	-

*Low=primary, lower vocational or lower secondary education; intermediate=intermediate vocational intermediate secondary or higher secondary education; high=higher vocational and university education. **Socio-economic status, classified as low, intermediate, and high based on a standard index including income, occupation and education per postal code area ('neighbourhood') obtained from Statistics Netherlands (www.cbs.nl). §Adjusted for all other variables in the table, except for neighbourhood SES, as it was collinear with the educational level and the inclusion of educational level in the model resulted in a better model fit (lower AIC) as compared to including neighbourhood SES. †Adjusted for all other variables in the table, except for educational level, as it was collinear with the neighbourhood SES and the inclusion of neighbourhood SES in the model resulted in a better model fit (lower AIC) as compared to including for educational level.

Supplementary Figures

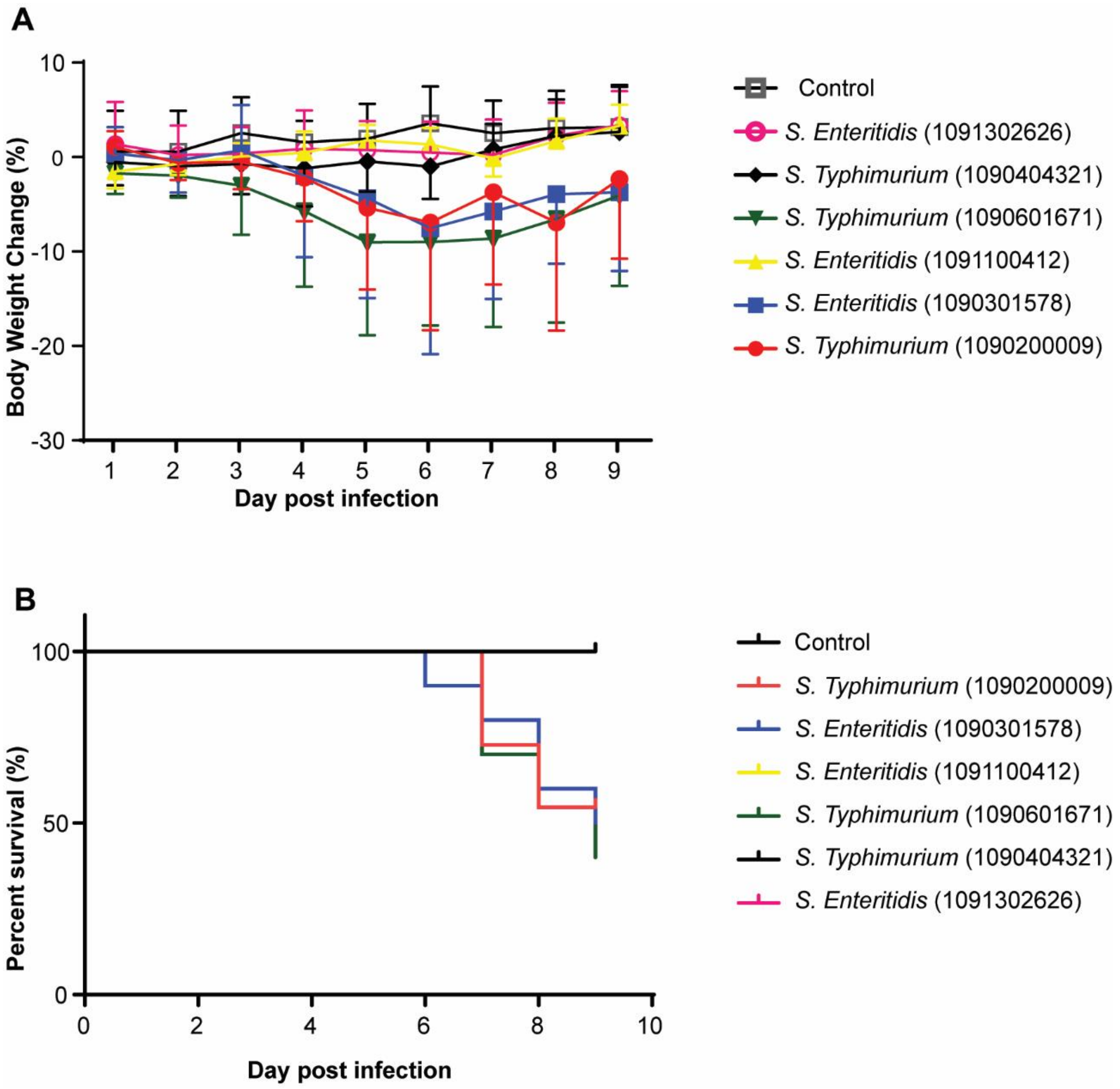

Supplementary Figure 1. Body weight and mortality of the clinical human NTS isolates. Related to Figure 2.

(A) Percent of body weight change throughout the experiment for indicated groups of the mice cohort. The animals (5-female and 5-male per groups) were inoculated with indicated NTS isolates (1×105 bacteria per mouse). Data was expressed by mean ± SD, n=10 mice each group. (B) Mortality of the infected animals throughout the experiment for indicated groups. n=10 mice each group.

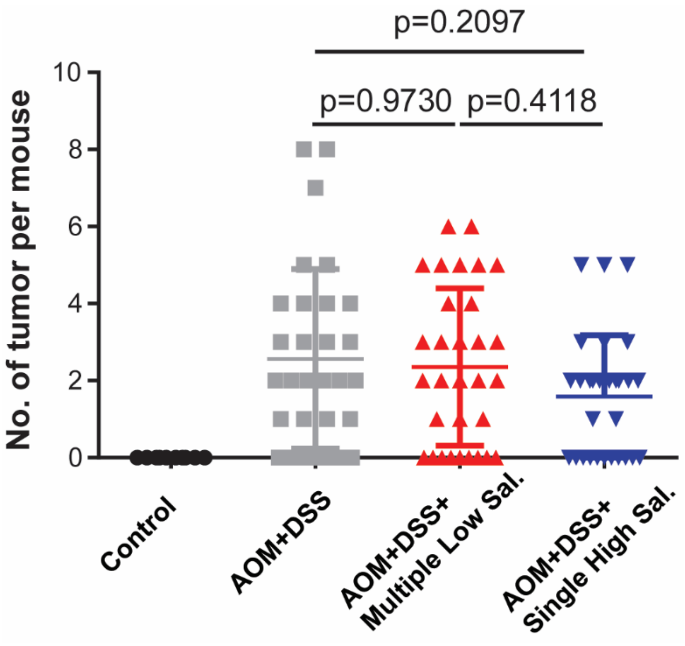

Supplementary Figure 2. Colonic tumors formed at a similar incidence throughout all treatment groups. Related to Figure 2.

The number of tumors per animal from each group was illustrated. n=10, 30, 31, and 29 for control, AOM+DSS, AOM+DSS+Multipe Low Sal. and AOM+DSS+Single High Sal. group, respectively. The data were expressed as mean ± SD; one-way ANOVA, and p-value as indicated in the figure.

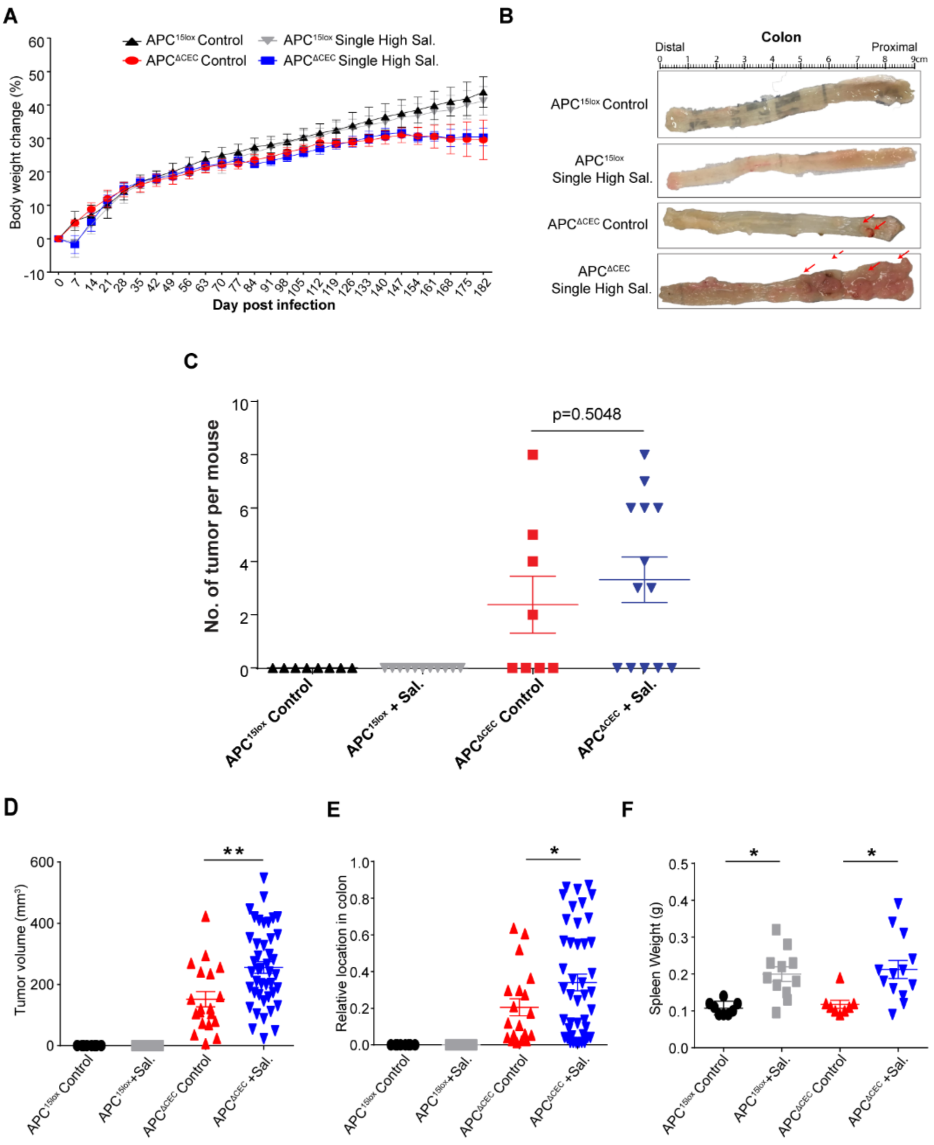

Supplementary Figure 3. NTS exposure increases tumor size and alter tumor location in spontaneous tumor mice model. Related to Figure 2.

(A) Percent of body weight change throughout the experiment for indicated groups of the mice cohort. Data were expressed as mean ± SD. Mice were infected with 10,000 CFU (100-µl suspension in HBSS) for one single high dose or treated with sterile HBSS as the control by oral gavage at day 1. n=8, 11, 8, and 13 for APC^{15lox} control, APC^{15lox} Single High Sal, APC$^{\Delta CEC}$ control and APC$^{\Delta CEC}$ Single High Sal. group, respectively. (***B***) Colonic tumors in situ. Representative colons of indicated groups of the mouse cohorts at 26 weeks post infection were illustrated. Tumors are indicated by red arrows. (***C***) The number of tumors per animal from each group was illustrated. n=8, 11, 8, and 13 for APC15lox control, APC15lox Single High Sal, APCΔCEC control, and APCΔCEC Single High Sal. group, respectively. The data were expressed as mean ± SD; one-way ANOVA, and p-value as indicated in the figure. (***D***) The tumor volume of indicated groups of within the mouse cohorts. The data was expressed as mean ±SD; one-way ANOVA, **p<0.01. (***E***) The tumor distribution of indicated groups within the mice cohort. The data was expressed as mean ± SD; Welch's t-test, *p<0.05. (***F***) The spleen weight of indicated groups within the mice cohort. The data was expressed as mean ± SD; one-way ANOVA, *p<0.05.

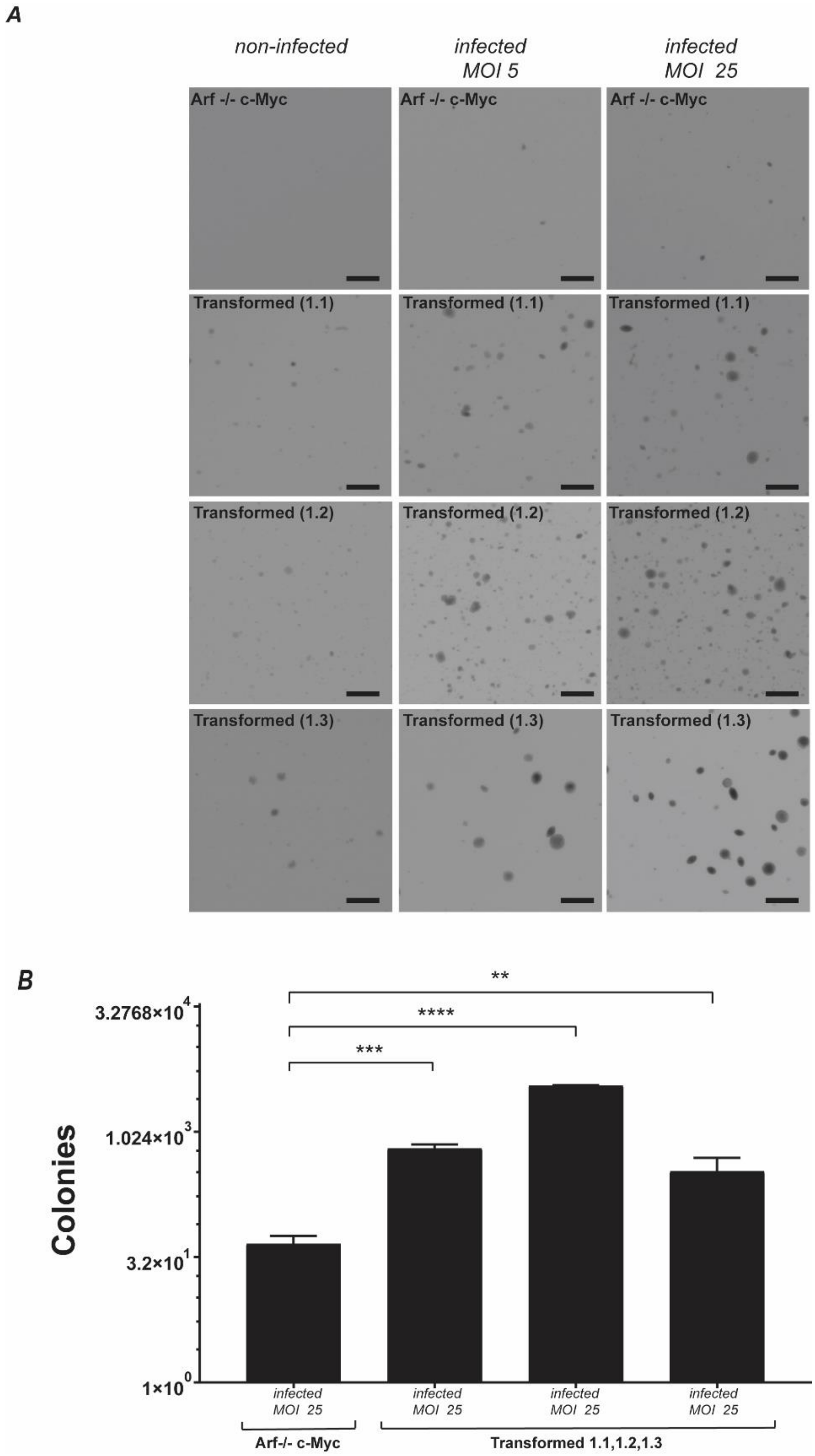

Supplementary Figure 4. Increased transformation upon repeated infections consistent amongst various NTS-transformed Arf-/- + c-Myc MEFs-isolates. Related to Figure 3.
(A) Representative images of anchorage-independent growth assays of Arf-/- c-Myc MEFs that have not previously encountered NTS (top panels: Arf-/- c-Myc) and of 3 Arf-/- c-Myc MEFs that did previously encountered NTS (bottom 3 panels; Transformed (1.1), (1.2) and (1.3)). Arf-/- c-Myc or transformed MEFs either non-infected, infected with an MOI of 5 or infected with an MOI of 25 are indicated in the left, middle and right panel, respectively. Scale bar: 750 µm. **(B)** Average number of soft agar colonies per well of naïve and transformed Arf-/- c-Myc MEFs that have been either non-infected, or infected with an MOI of 25. The data was expressed as mean ± SD; one-way ANOVA, ****$p<0.0001$, ***$p<0.001$, **$p<0.01$. Results derive from two independent experiments with technical triplicates.

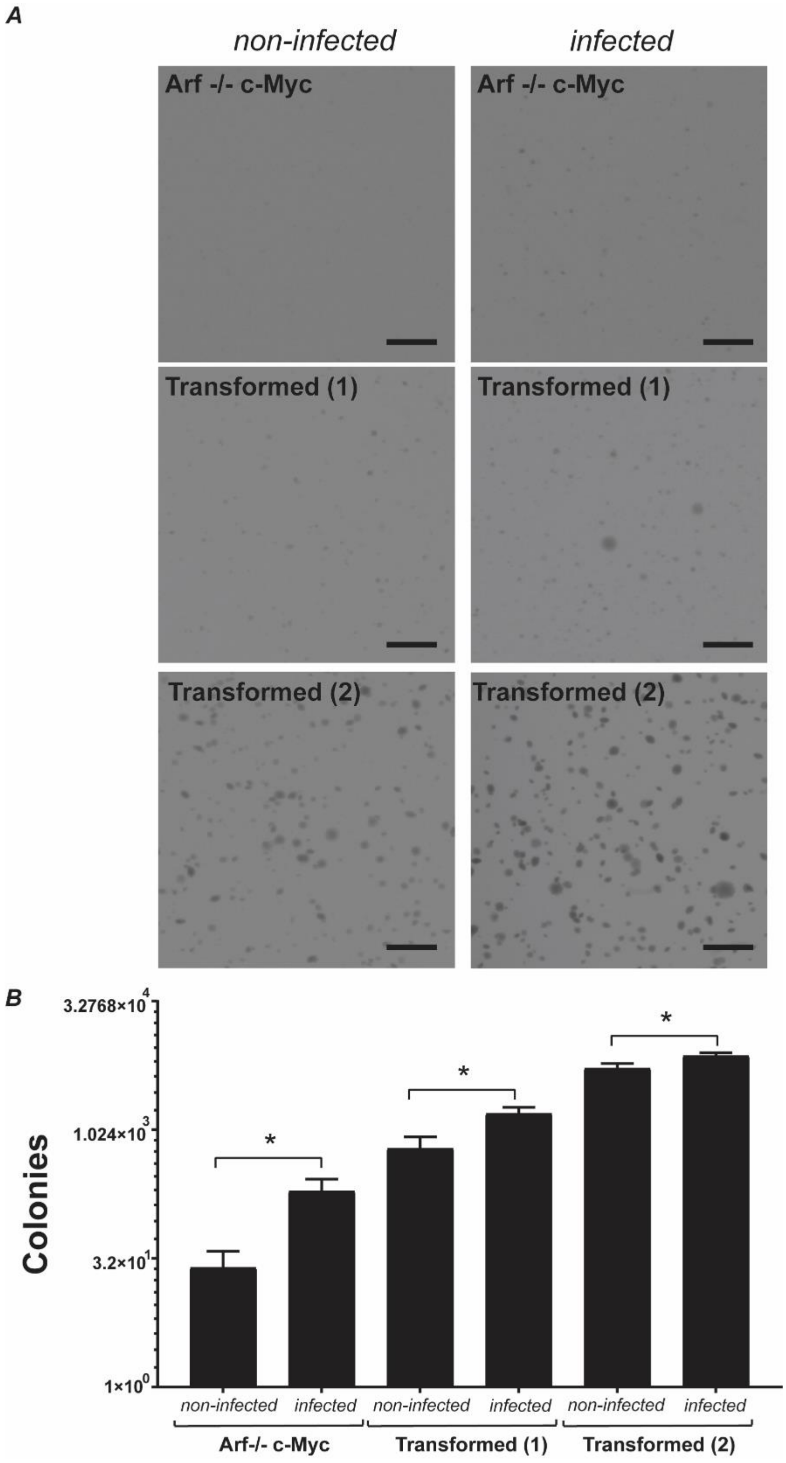

Supplementary Figure 5. Three successive rounds of NTS infection further increases transformation. Related to Figure 3.

(**A**) Representative images of anchorage-independent growth assays of Arf-/- c-Myc MEFs that have not previously encountered NTS (top row: Arf-/- c-Myc), that have previously encountered NTS once (middle row: Transformed (1)), and that have previously encountered NTS twice (bottom row). Arf-/- c-Myc or transformed MEFs non-infected are placed in the left column and Arf-/- c-Myc or transformed MEFs infected with NTS (MOI 25) are placed and right column, as indicated. Scale bar: 750 µm. **(B)** Average number of soft agar colonies per well of naïve and transformed Arf-/- c-Myc MEFs that have been either non-infected, or infected with an MOI of 25. Data was expressed as mean ± SD experiments; one-way ANOVA, *p<0.1. Results derive from two independent experiments with technical triplicates.

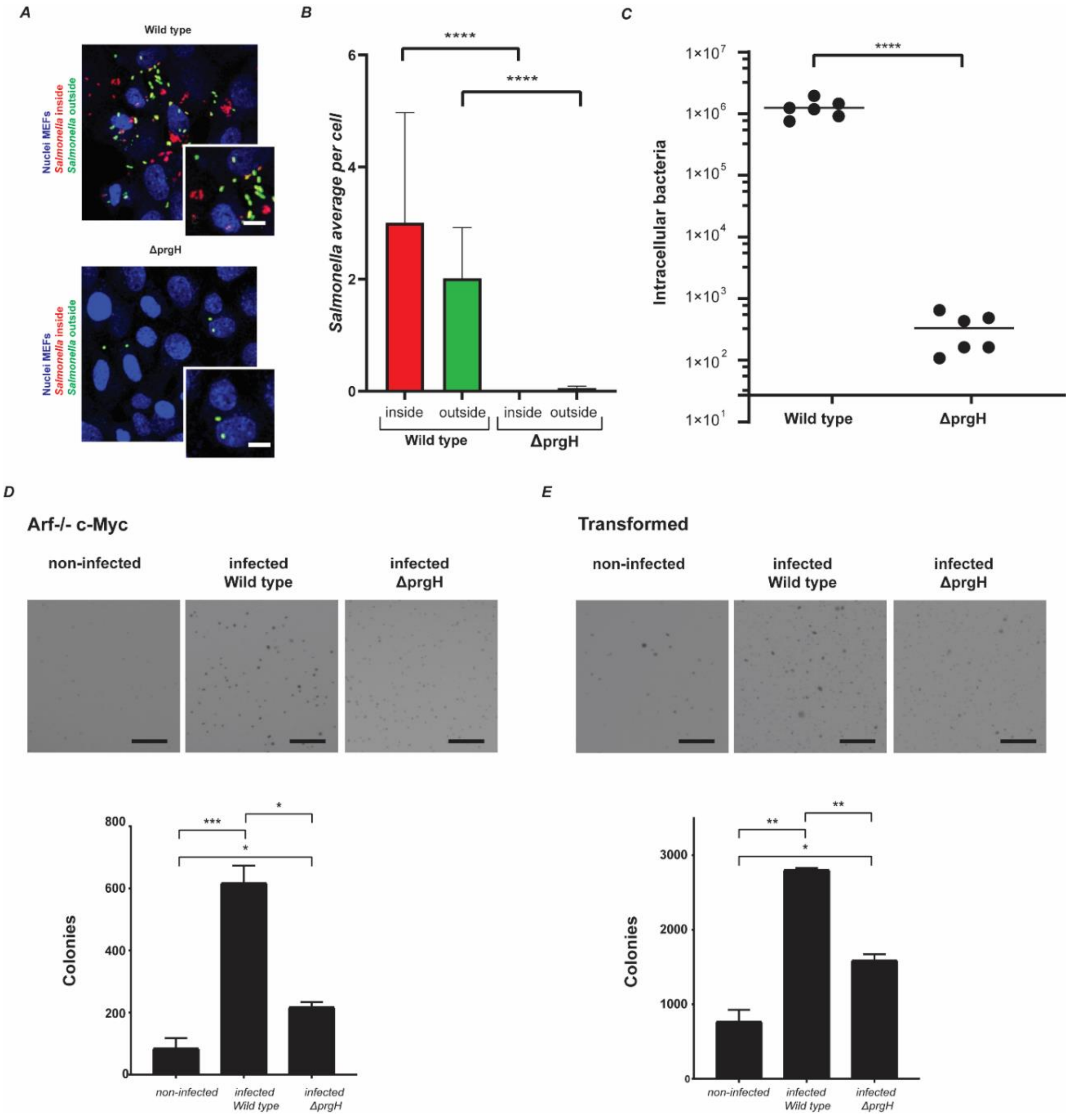

Supplementary Figure 6. Infection of transformed MEFs with NTS ΔprgH averts increased host cell adherence infection and limits the transformative effect of an initial and recurrent NTS infection. Related to Figure 3.

(**A**) Representative images of intra-(inside) and extracellular (outside) NTS bacteria in transformed MEFs after infection with NTS Wild type (upper) and NTS (ΔprgH) at MOI 25 for 1 hour. Scale bar 10 μm. **(B)** Quantification of intra-(inside) and extracellular (outside) NTS bacteria in transformed MEFs after infection with NTS Wild type (upper) and NTS (ΔprgH) at MOI 25 for 1 hour. Data was expressed as mean ± SD; one-way ANOVA, ****p<0.0001. **(C)** CFU counts of intracellular Wild type and ΔprgH bacteria in transformed MEFs after infection at MOI 25 for 1 hour. Data was expressed as mean ± SD; one-way ANOVA, ****p<0.0001. (**D**) Representative images of anchorage-independent growth assays of Arf-/- c-Myc MEFs that have not previously encountered NTS either non-infected (left), infected with Wild type NTS (middle) or NTS ΔprgH (right) at MOI 25 for 1 hour, and average number of soft agar colonies per well of naïve Arf-/- c-Myc MEFs either non-infected (left), infected with Wild type NTS (middle) and NTS ΔprgH (right) at MOI 25 for 1 hour. Data was expressed as mean ± SD; one-way ANOVA, ***p<0.001, *p<0.1. (**E**) Representative images of anchorage-independent growth assays of Transformed Arf-/- c-Myc MEFs either non-infected (left), infected with Wild type NTS (middle) or NTS ΔprgH (right) at MOI 25 for 1 hour, and average number of soft agar colonies per well Transformed MEFs either non-infected (left), infected with Wild type NTS (middle) or NTS ΔprgH (right) at MOI 25 for 1 hour. Data was expressed as mean ± SD; one-way ANOVA, **p<0.01, *p<0.1. Results derive from two independent experiments with technical triplicates.

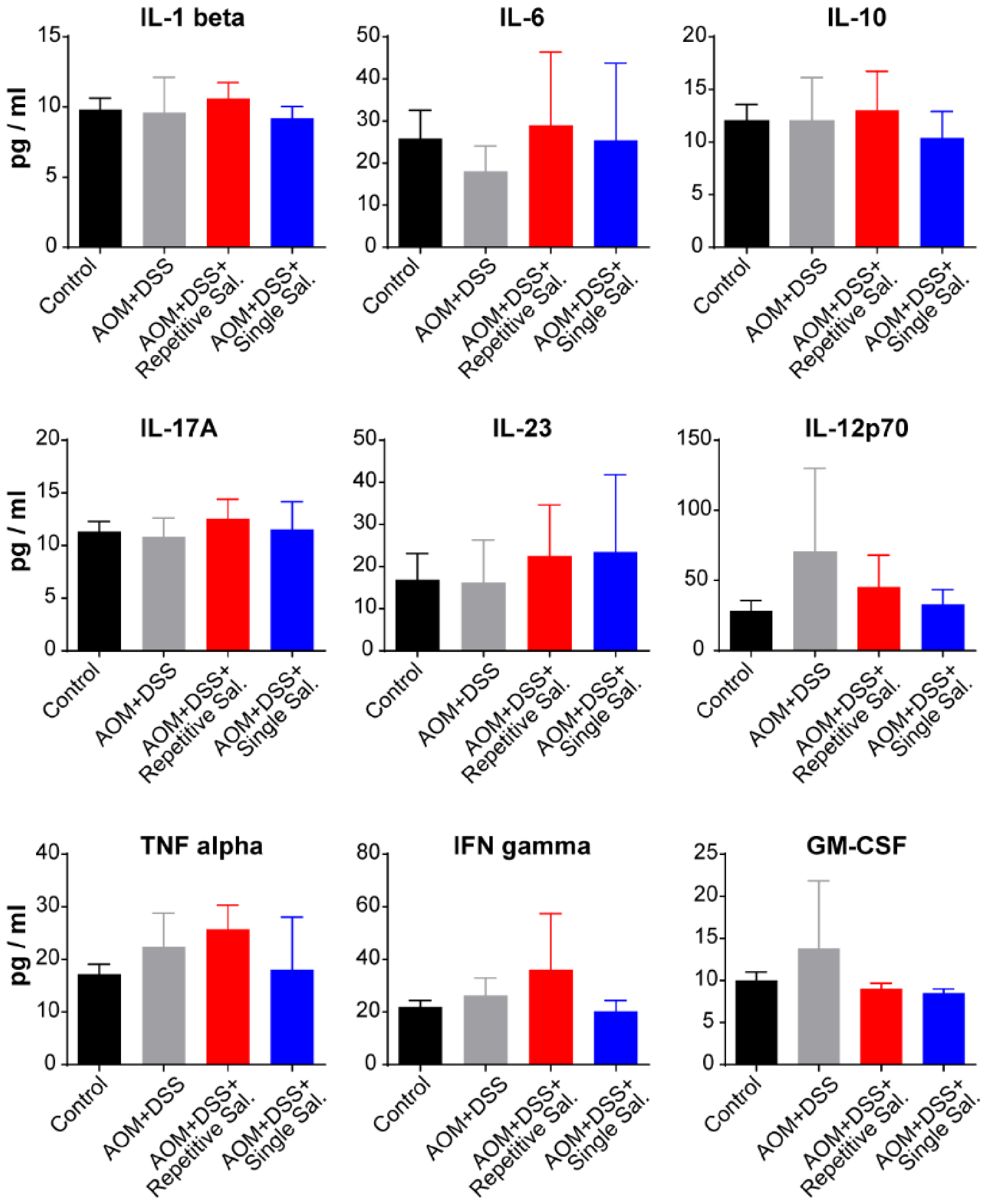

Supplementary Figure 7: Cytokine and chemokines in plasma from the AOM+DSS mice model either or not infected with NTS. Related to Figure 2.

The cytokines and chemokines in the serum from the experimental animals were evaluated with the Luminex kit according to product's instructions. The data was expressed as mean+SD; one-way ANOVA, n=5 per group.
